# Crystal structure of 4,5,6,7,8,8-hexa­chloro-2-(3,4-di­meth­oxy­pheneth­yl)-3a,4,7,7a-tetra­hydro-1*H*-4,7-methano­iso­indole-1,3(2*H*)-dione [+solvent]

**DOI:** 10.1107/S2056989019004109

**Published:** 2019-04-02

**Authors:** R. Manohar, M. Harikrishna, S. Harikrishna Etti, C. Ramanathan, K. Gunasekaran

**Affiliations:** a Centre of Advanced Study in Crystallography and Biophysics, University of Madras, Guindy Campus, Chennai 600 025, India; b Department of Chemistry, Pondicherry University, Pondicherry 605 014, India; c Department of Medical Physics, Bharathiar University, Coimbatore, India

**Keywords:** crystal structure, norbornene, hexa­chloro, iso­indolene, hydrogen bonding, offset π–π inter­action, C—Cl⋯π inter­action

## Abstract

In the title compound, C_19_H_15_Cl_6_NO_4_ [+solvent], the six-membered ring of the norbornene moiety adopts a boat conformation and the two five-membered rings have envelope conformations. The pyrrolidine ring makes a dihedral angle of 14.83 (12)° with the 3,4-di­meth­oxy­phenyl ring, which are attached to each other by an extended N—CH_2_—CH_2_—C_ar_ bridge. In the crystal, the structure features C—H⋯O inter­molecular hydrogen bonds, an offset π–π inter­action [inter­centroid distance = 3.564 (1) Å] and a C—Cl⋯π inter­action. The contribution of some disordered solvent to the scattering was removed using the SQUEEZE routine [Spek (2015 ▸). Acta Cryst. C71, 9–18] of *PLATON*. The solvent contribution was not included in the reported mol­ecular weight and density.

## Abstract 2

In the title compound, the pyrrolidine ring makes a dihedral angle of 14.83 (12)° with the 3,4-di­meth­oxy­phenyl ring, which are attached to each other by an extended N—CH_2_—CH_2_—C_ar_ bridge.

## Chemical context   

One of the fundamental objectives of organic and medicinal chemistry is the design and synthesis of mol­ecules having value as human therapeutic agents (Patil & Rajput, 2014 ▸). Succinimide derivatives are significant compounds found in various natural products, and have outstanding biological and pharmaceutical activity (Ahire & Mhaske, 2017 ▸). Cyclic imides and their derivatives contain an imide ring and the general structure –CO–*-*N(*R*)—CO–, and can cross biological membranes *in vivo* (Hargreaves *et al.*, 1970 ▸). The variety of biological activities and pharmaceutical uses of compounds containing a succinimide moiety is considerable. They include activities such as anti­fungal (Hazra *et al.*, 2004 ▸), anti-tubercular (Isaka *et al.*, 2006 ▸), CNS depressant (Aeberli *et al.*, 1976 ▸), anti­spasmodic (Nunes *et al.*, 1995 ▸), cytostatic (Crider *et al.*, 1980 ▸), analgesic (Correa *et al.*, 1997 ▸), anti­bacterial (Zentz *et al.*, 2002 ▸), anti­cancer (Hall *et al.*, 1995 ▸), anorectic (Rich & Gardner, 1983 ▸), hypotensive (Coram & Brezenoff, 1983 ▸), nerve conduction blocking (Kaczorowski *et al.*, 2008 ▸), bacteriostatic (Piper *et al.*, 1971 ▸), anti-convulsant (Kornet *et al.*, 1977 ▸) and muscle relaxant (Musso *et al.*, 2003 ▸).
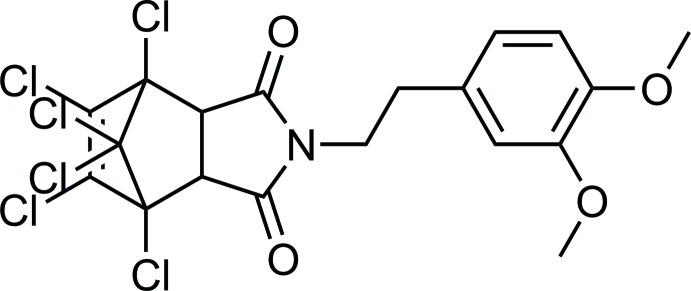


## Structural commentary   

**Figure 1:**
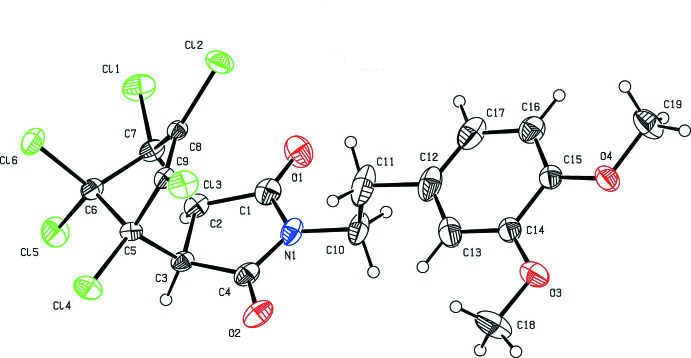
The mol­ecular structure of the title compound, with the atom labelling. Displacement ellipsoids are drawn at 30% probability level.

The mol­ecular structure of the title compound is shown in Fig. 1 ▸. The six-membered ring of the norbornene moiety (C2/C3/C5/C7–C9) adopts a boat conformation [puckering parameters: amplitude *Q* = 0.939 (2) Å, θ = 90.00 (12)°, φ = 299.27 (14)°]. The two five-membered rings, *A* (C2/C3/C5–C7) and *B* (C5–C9), have envelope conformations with atom C6 as the flap: puckering parameters and the smallest displacement asymmetric parameters are *Q*_2_ = 0.619 (2) Å, φ_2_ = 108.6 (2)° and Δ*s* = 1.09° for ring *A*, and *Q*_2_ = 0.582 (2) Å, φ_2_ = 215.5 (2)° and Δ*s* = 0.74° for ring *B*. Atom C6 is displaced from the mean plane through the other four atoms by 0.908 (2) Å in ring *A* and 0.875 (2) Å in ring *B*. The dihedral angle between the pyrrolidine ring (N1/C1–C4) and the benzene ring (C12–C17) is 14.83 (12)°, with the torsion angle N1—C10—C11—C12 being 175.8 (3)°. The lengths of the C—Cl bonds involving the chlorine atoms attached to the C8=C9 double bond are 1.692 (2) Å for C8—Cl2 and 1.692 (2) Å for C9—Cl3. The lengths of the bonds to chlorine atoms attached to the single C—C bonds vary from 1.744 (2) to 1.768 (2) Å. These value are close to those found in similar compounds; see §4 *Database survey.*

## Supra­molecular features   

**Figure 2:**
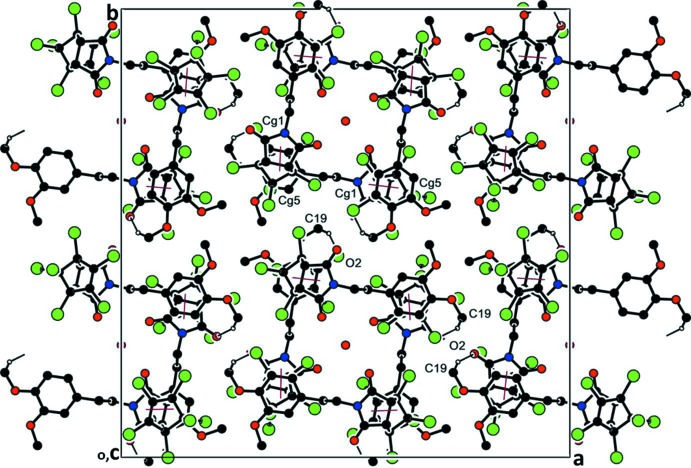
A view along the *c* axis of the crystal packing of the title compound. The C—H⋯O hydrogen bonds (thin black lines; Table 1 ▸) generate an 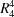
(48) ring motif. The offset π–π inter­action is shown as a thin red line. For clarity, H atoms not involved in hydrogen bonding have been omitted.

In the crystal, weak C19—H19*A*⋯O2^i^ hydrogen bonds link the molecules to form a cyclic 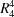
(48) ring motif (Table 1 ▸ and Fig. 2 ▸). The mol­ecules are stacked in layers held together by offset π–π inter­actions (Fig. 2 ▸), with an inter­centroid distance *Cg*1⋯*Cg*5^iii^ of 3.564 (1) Å [*Cg*1 and *Cg*5 are the centroids of the pyrrolidine (N1/C1–C4) and benzene (C12–C17) rings, respectively, α = 9.80 (12)°, inter­planar distances are 3.448 (1) and 3.547 (1) Å, offset = 0.353 Å; symmetry code: (iii) −*y* + 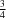, *x* − 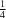, −*z* + 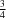]. There is also an inter­molecular C—Cl⋯π inter­action present, involving atom Cl6 and the centroid of the benzene ring (C12–C17); see Table 1 ▸.

## Database survey   

A search of the Cambridge Structural Database (CSD, V 5.40, update February 2019; Groom *et al.*, 2016 ▸) for the 4,5,6,7,8,8-hexa­chloro-3a,4,7,7a-tetra­hydro-1*H*-4,7-methano­iso­indole-1,3(2*H*)-dione skeleton yielded 17 hits (see supporting information). The majority of these compounds have thio­phene substituents. One compound, 1,7,8,9,10,10-hexa­chloro-4-(2-phenyl­eth­yl)-4-aza­tri­cyclo­[5.2.1.0^2,6^]dec-8-ene-3,5-dione (CSD refcode EVEDIT; Manohar *et al.*, 2011 ▸), closely resembles the title compound but has a 2-phenethyl substit­uent rather than the 2-(3,4-di­methyl­pheneth­yl) group in the title compound. Here, the aryl ring is inclined to the pyrrolidine ring by 7.43 (16)° compared to 14.83 (12)° in the title compound, and the N—C—C—C_ar_ torsion angle is −169.3 (3)° compared to 175.8 (3)° in the title compound.

In all 17 structures, the five-membered ring has envelope conformations and the six-membered ring a boat conformation. The bond lengths and bond angles are very similar to those reported here for the title compound. For example, the C*sp* ^2^—Cl bond lengths are shorter than the C*sp* ^3^—Cl bond lengths; the former vary from *ca* 1.681 to 1.717 Å, while the latter vary from *ca* 1.725 to 1.798 Å. In the title compound these bond lengths are 1.691 (2)–1.692 (2) Å and 1.744 (2)–1.768 (2) Å, respectively.

## Synthesis and crystallization   

2-(3,4-Di­meth­oxy­phen­yl) ethanamine (1 equiv.) and 1,4,5,6,7,7-hexa­chloro-5- norbornene −2,3-di­carb­oxy­lic anhydride (1 equiv.) were stirred at room temperature in dry ethyl acetate for 30 min. The ethyl acetate was removed under reduced pressure and the resulting residue was dissolved in toluene. To this reaction mixture was added acetyl chloride (5 equiv.) and refluxed for 1 h. The reaction mixture was brought to room temperature and washed with aqueous Na_2_CO_3_ and dried over anhydrous Na_2_SO_4_. It was then filtered and the filtrate was concentrated under reduced pressure followed by silica gel column purification to afford the title compound in 82% yield. Colourless block-shaped crystals were obtained by slow evaporation of a solution in ethanol.

## Refinement   

Crystal data, data collection and structure refinement details are summarized in Table 2 ▸. The hydrogen atoms were placed in calculated positions and refined using a riding model: C—H = 0.93–0.98 Å with *U*_iso_(H) = 1.5*U*_eq_(C-meth­yl) and 1.2*U*_eq_(C) for other H atoms. The contribution of the disordered solvent to the scattering was removed using the SQUEEZE routine of *PLATON* (Spek, 2015 ▸). The solvent contribution was not included in the reported mol­ecular weight and density. Further details are given in the archived CIF.

## Supplementary Material

Crystal structure: contains datablock(s) Global, I. DOI: 10.1107/S2056989019004109/su5485sup1.cif

Structure factors: contains datablock(s) I. DOI: 10.1107/S2056989019004109/su5485Isup3.hkl

CSD search S1. DOI: 10.1107/S2056989019004109/su5485sup4.pdf

Supporting information file. DOI: 10.1107/S2056989019004109/su5485Isup4.cml

CCDC reference: 1905872

Additional supporting information:  crystallographic information; 3D view; checkCIF report

## Figures and Tables

**Table 1:** Hydrogen-bond geometry (Å, °) *Cg*5 is the centroid of the C12–C17 benzene ring.

*D*—H⋯*A*	*D*—H	H⋯*A*	*D*⋯*A*	*D*—H⋯*A*
C19—H19*A*⋯O2^i^	0.96	2.57	3.408 (4)	146
C6—Cl6⋯*Cg*5^ii^	1.77 (1)	3.41 (1)	4.894 (2)	140 (1)

Symmetry codes: (i) 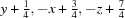; (ii) 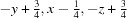.

**Table 2:** Experimental details

Crystal data	
Chemical formula	C_19_H_15_Cl_6_NO_4_[+solvent]
*M*_r_	534.02
Crystal system, space group	Tetragonal, *I*4_1_/*a*
Temperature (K)	293
*a*, *c* (Å)	29.6250 (9), 10.2427 (4)
*V* (Å^3^)	8989.4 (6)
*Z*	16
Radiation type	Mo *K*α
μ (mm^−1^)	0.79
Crystal size (mm)	0.26 × 0.21 × 0.15

Data collection	
Diffractometer	Bruker SMART APEXII area-detector
Absorption correction	Multi-scan (*SADABS*; Bruker, 2008 ▸)
*T*_min_, *T*_max_	0.752, 0.863
No. of measured, independent and observed [*I* > 2σ(*I*)] reflections	10728, 5181, 3330
*R*_int_	0.021
(sin θ/λ)_max_ (Å^−1^)	0.687

Refinement	
*R*[*F* ^2^ > 2σ(*F* ^2^)], *wR*(*F* ^2^), *S*	0.041, 0.101, 1.04
No. of reflections	5181
No. of parameters	273
H-atom treatment	H-atom parameters constrained
Δρ_max_, Δρ_min_ (e Å^−3^)	0.33, −0.21

Computer programs: *APEX2* and *SAINT* (Bruker, 2008 ▸), *SHELXS97* (Sheldrick, 2008 ▸), *ORTEP-3 for Windows* (Farrugia, 2012 ▸), *SHELXL2018* (Sheldrick, 2015 ▸) and *PLATON* (Spek, 2009 ▸).

## supplementary crystallographic information

### Crystal data

**Table:**

C_19_H_15_Cl_6_NO_4_[+solvent]	*D*_x_ = 1.578 Mg m^−^^3^
*M**_r_* = 534.02	Mo *K*α radiation, λ = 0.71073 Å
Tetragonal, *I*4_1_/*a*	Cell parameters from 5181 reflections
*a* = 29.6250 (9) Å	θ = 2.8–29.2°
*c* = 10.2427 (4) Å	µ = 0.79 mm^−^^1^
*V* = 8989.4 (6) Å^3^	*T* = 293 K
*Z* = 16	Block, colourless
*F*(000) = 4320	0.26 × 0.21 × 0.15 mm

### Data collection

**Table:**

Bruker SMART APEXII area-detector diffractometer	3330 reflections with *I* > 2σ(*I*)
ω and φ scans	*R*_int_ = 0.021
Absorption correction: multi-scan (*SADABS*; Bruker, 2008)	θ_max_ = 29.2°, θ_min_ = 2.8°
*T*_min_ = 0.752, *T*_max_ = 0.863	*h* = −32→24
10728 measured reflections	*k* = −40→24
5181 independent reflections	*l* = −12→12

### Refinement

**Table:**

Refinement on *F*^2^	Primary atom site location: structure-invariant direct methods
Least-squares matrix: full	Secondary atom site location: difference Fourier map
*R*[*F*^2^ > 2σ(*F*^2^)] = 0.041	Hydrogen site location: inferred from neighbouring sites
*wR*(*F*^2^) = 0.101	H-atom parameters constrained
*S* = 1.04	*w* = 1/[σ^2^(*F*_o_^2^) + (0.046*P*)^2^ + 2.6033*P*] where *P* = (*F*_o_^2^ + 2*F*_c_^2^)/3
5181 reflections	(Δ/σ)_max_ = 0.001
273 parameters	Δρ_max_ = 0.33 e Å^−^^3^
0 restraints	Δρ_min_ = −0.21 e Å^−^^3^

### Special details

**Table:**

Geometry. All esds (except the esd in the dihedral angle between two l.s. planes) are estimated using the full covariance matrix. The cell esds are taken into account individually in the estimation of esds in distances, angles and torsion angles; correlations between esds in cell parameters are only used when they are defined by crystal symmetry. An approximate (isotropic) treatment of cell esds is used for estimating esds involving l.s. planes.

### Fractional atomic coordinates and isotropic or equivalent isotropic displacement parameters (Å^2^)

**Table:**

	*x*	*y*	*z*	*U*_iso_*/*U*_eq_	
Cl1	0.64197 (3)	0.18131 (2)	0.50159 (7)	0.0609 (2)	
Cl2	0.53878 (2)	0.16505 (2)	0.39238 (6)	0.05182 (19)	
Cl3	0.51735 (2)	0.05095 (2)	0.39790 (6)	0.04926 (18)	
Cl4	0.60427 (3)	−0.00099 (2)	0.52552 (7)	0.0560 (2)	
Cl5	0.68772 (2)	0.07881 (3)	0.59849 (7)	0.0604 (2)	
Cl6	0.65299 (2)	0.08158 (2)	0.33757 (6)	0.05024 (19)	
O1	0.55317 (7)	0.18699 (7)	0.73520 (19)	0.0638 (6)	
O2	0.51935 (7)	0.03730 (7)	0.74848 (17)	0.0583 (5)	
O3	0.29789 (7)	0.07109 (6)	0.78565 (19)	0.0606 (5)	
O4	0.26332 (6)	0.14834 (6)	0.75279 (18)	0.0512 (5)	
N1	0.52660 (7)	0.11436 (8)	0.74800 (18)	0.0436 (5)	
C1	0.55897 (9)	0.14695 (10)	0.7253 (2)	0.0452 (6)	
C2	0.60172 (8)	0.12358 (8)	0.6826 (2)	0.0374 (6)	
H2	0.627206	0.131273	0.739349	0.045*	
C3	0.59071 (7)	0.07269 (8)	0.6881 (2)	0.0345 (5)	
H3	0.610647	0.056594	0.748616	0.041*	
C4	0.54179 (9)	0.07055 (10)	0.7313 (2)	0.0419 (6)	
C5	0.59788 (8)	0.05717 (7)	0.5446 (2)	0.0321 (5)	
C6	0.63858 (7)	0.08698 (9)	0.5043 (2)	0.0359 (6)	
C7	0.61330 (8)	0.13120 (8)	0.5366 (2)	0.0353 (5)	
C8	0.57030 (7)	0.12315 (8)	0.45795 (19)	0.0304 (5)	
C9	0.56157 (7)	0.07940 (8)	0.46217 (19)	0.0291 (5)	
C10	0.47983 (9)	0.12501 (11)	0.7835 (2)	0.0561 (8)	
H10A	0.478774	0.154042	0.827179	0.067*	
H10B	0.468475	0.102335	0.843401	0.067*	
C11	0.45025 (10)	0.12635 (15)	0.6621 (3)	0.0885 (13)	
H11A	0.460751	0.150372	0.605383	0.106*	
H11B	0.453546	0.098090	0.615245	0.106*	
C12	0.40091 (10)	0.13382 (14)	0.6918 (3)	0.0630 (9)	
C13	0.37364 (10)	0.09809 (11)	0.7279 (2)	0.0580 (8)	
H13	0.386380	0.069607	0.737894	0.070*	
C14	0.32777 (9)	0.10361 (9)	0.7495 (2)	0.0439 (6)	
C15	0.30858 (8)	0.14660 (9)	0.7326 (2)	0.0396 (6)	
C16	0.33546 (9)	0.18220 (10)	0.6993 (2)	0.0519 (7)	
H16	0.322956	0.210806	0.689646	0.062*	
C17	0.38163 (10)	0.17583 (13)	0.6796 (3)	0.0641 (9)	
H17	0.399661	0.200407	0.657959	0.077*	
C18	0.31333 (14)	0.02580 (11)	0.7890 (4)	0.0954 (13)	
H18A	0.323689	0.017167	0.703745	0.143*	
H18B	0.289057	0.006337	0.815147	0.143*	
H18C	0.337711	0.023232	0.850281	0.143*	
C19	0.24021 (10)	0.18878 (10)	0.7231 (3)	0.0670 (9)	
H19A	0.249922	0.212192	0.781535	0.101*	
H19B	0.208313	0.184150	0.732920	0.101*	
H19C	0.246680	0.197481	0.634816	0.101*	

### Atomic displacement parameters (Å^2^)

**Table:**

	*U*^11^	*U*^22^	*U*^33^	*U*^12^	*U*^13^	*U*^23^
Cl1	0.0617 (5)	0.0460 (4)	0.0751 (5)	−0.0269 (4)	0.0008 (4)	0.0089 (3)
Cl2	0.0569 (4)	0.0473 (4)	0.0512 (4)	0.0131 (3)	−0.0095 (3)	0.0098 (3)
Cl3	0.0436 (4)	0.0551 (4)	0.0491 (4)	−0.0167 (3)	−0.0143 (3)	−0.0023 (3)
Cl4	0.0691 (5)	0.0336 (3)	0.0652 (4)	0.0060 (3)	0.0037 (4)	0.0058 (3)
Cl5	0.0315 (3)	0.0879 (6)	0.0618 (4)	0.0052 (4)	−0.0120 (3)	0.0078 (4)
Cl6	0.0448 (4)	0.0656 (5)	0.0404 (3)	−0.0024 (3)	0.0122 (3)	0.0030 (3)
O1	0.0729 (15)	0.0533 (13)	0.0651 (13)	0.0064 (11)	−0.0032 (11)	−0.0172 (10)
O2	0.0576 (12)	0.0645 (13)	0.0527 (11)	−0.0226 (11)	0.0096 (9)	0.0062 (10)
O3	0.0659 (13)	0.0456 (11)	0.0703 (13)	0.0102 (11)	−0.0031 (11)	0.0087 (10)
O4	0.0419 (11)	0.0452 (10)	0.0664 (12)	0.0106 (9)	−0.0006 (9)	0.0052 (9)
N1	0.0363 (12)	0.0636 (15)	0.0310 (10)	−0.0031 (11)	0.0025 (9)	−0.0060 (10)
C1	0.0504 (17)	0.0571 (17)	0.0282 (12)	−0.0019 (15)	−0.0080 (11)	−0.0082 (12)
C2	0.0345 (13)	0.0465 (15)	0.0312 (12)	−0.0078 (11)	−0.0062 (10)	−0.0023 (11)
C3	0.0310 (12)	0.0449 (14)	0.0275 (11)	−0.0035 (11)	−0.0047 (10)	0.0076 (10)
C4	0.0439 (15)	0.0561 (17)	0.0258 (12)	−0.0064 (14)	−0.0029 (11)	0.0023 (12)
C5	0.0362 (13)	0.0293 (12)	0.0309 (11)	−0.0027 (10)	−0.0018 (10)	0.0053 (10)
C6	0.0280 (12)	0.0471 (15)	0.0326 (12)	−0.0034 (11)	−0.0031 (10)	0.0063 (11)
C7	0.0341 (13)	0.0352 (13)	0.0366 (12)	−0.0143 (11)	−0.0020 (10)	0.0025 (11)
C8	0.0272 (12)	0.0397 (13)	0.0245 (10)	−0.0003 (10)	−0.0018 (9)	0.0029 (10)
C9	0.0261 (11)	0.0379 (13)	0.0234 (10)	−0.0044 (10)	−0.0026 (9)	−0.0002 (10)
C10	0.0403 (15)	0.089 (2)	0.0392 (14)	0.0055 (15)	0.0103 (12)	−0.0088 (14)
C11	0.0425 (17)	0.181 (4)	0.0419 (17)	0.017 (2)	0.0052 (14)	−0.009 (2)
C12	0.0434 (17)	0.115 (3)	0.0300 (14)	0.006 (2)	0.0000 (12)	−0.0106 (16)
C13	0.0549 (18)	0.086 (2)	0.0331 (14)	0.0287 (18)	−0.0063 (13)	−0.0089 (14)
C14	0.0463 (16)	0.0576 (17)	0.0278 (12)	0.0119 (14)	−0.0041 (11)	−0.0027 (12)
C15	0.0411 (15)	0.0490 (15)	0.0288 (12)	0.0042 (13)	−0.0035 (11)	−0.0023 (11)
C16	0.0534 (18)	0.0598 (18)	0.0426 (14)	−0.0016 (15)	0.0002 (13)	−0.0013 (14)
C17	0.0541 (19)	0.094 (3)	0.0438 (16)	−0.0127 (19)	0.0066 (14)	−0.0035 (17)
C18	0.114 (3)	0.056 (2)	0.116 (3)	0.029 (2)	−0.003 (3)	0.021 (2)
C19	0.0515 (18)	0.0576 (19)	0.092 (2)	0.0157 (16)	−0.0160 (17)	0.0047 (17)

### Geometric parameters (Å, º)

**Table:**

Cl1—C7	1.748 (2)	C6—C7	1.545 (3)
Cl2—C8	1.692 (2)	C7—C8	1.526 (3)
Cl3—C9	1.691 (2)	C8—C9	1.322 (3)
Cl4—C5	1.744 (2)	C10—C11	1.522 (4)
Cl5—C6	1.763 (2)	C10—H10A	0.9700
Cl6—C6	1.768 (2)	C10—H10B	0.9700
O1—C1	1.203 (3)	C11—C12	1.509 (4)
O2—C4	1.201 (3)	C11—H11A	0.9700
O3—C14	1.360 (3)	C11—H11B	0.9700
O3—C18	1.418 (3)	C12—C17	1.375 (4)
O4—C15	1.358 (3)	C12—C13	1.382 (4)
O4—C19	1.413 (3)	C13—C14	1.386 (4)
N1—C1	1.380 (3)	C13—H13	0.9300
N1—C4	1.384 (3)	C14—C15	1.405 (4)
N1—C10	1.467 (3)	C15—C16	1.365 (4)
C1—C2	1.508 (3)	C16—C17	1.395 (4)
C2—C3	1.543 (3)	C16—H16	0.9300
C2—C7	1.550 (3)	C17—H17	0.9300
C2—H2	0.9800	C18—H18A	0.9600
C3—C4	1.516 (3)	C18—H18B	0.9600
C3—C5	1.554 (3)	C18—H18C	0.9600
C3—H3	0.9800	C19—H19A	0.9600
C5—C9	1.518 (3)	C19—H19B	0.9600
C5—C6	1.551 (3)	C19—H19C	0.9600
			
C14—O3—C18	117.8 (2)	C8—C9—Cl3	128.85 (18)
C15—O4—C19	118.6 (2)	C5—C9—Cl3	123.33 (17)
C1—N1—C4	114.1 (2)	N1—C10—C11	110.3 (2)
C1—N1—C10	123.2 (2)	N1—C10—H10A	109.6
C4—N1—C10	122.6 (2)	C11—C10—H10A	109.6
O1—C1—N1	125.2 (3)	N1—C10—H10B	109.6
O1—C1—C2	126.7 (3)	C11—C10—H10B	109.6
N1—C1—C2	108.1 (2)	H10A—C10—H10B	108.1
C1—C2—C3	105.1 (2)	C12—C11—C10	113.4 (2)
C1—C2—C7	113.51 (19)	C12—C11—H11A	108.9
C3—C2—C7	102.98 (18)	C10—C11—H11A	108.9
C1—C2—H2	111.6	C12—C11—H11B	108.9
C3—C2—H2	111.6	C10—C11—H11B	108.9
C7—C2—H2	111.6	H11A—C11—H11B	107.7
C4—C3—C2	104.7 (2)	C17—C12—C13	118.3 (3)
C4—C3—C5	113.19 (18)	C17—C12—C11	121.1 (4)
C2—C3—C5	103.01 (17)	C13—C12—C11	120.5 (3)
C4—C3—H3	111.8	C12—C13—C14	121.7 (3)
C2—C3—H3	111.8	C12—C13—H13	119.2
C5—C3—H3	111.8	C14—C13—H13	119.2
O2—C4—N1	124.8 (2)	O3—C14—C13	126.7 (3)
O2—C4—C3	127.3 (3)	O3—C14—C15	114.3 (2)
N1—C4—C3	107.9 (2)	C13—C14—C15	118.9 (3)
C9—C5—C6	98.95 (17)	O4—C15—C16	125.8 (2)
C9—C5—C3	107.51 (18)	O4—C15—C14	114.5 (2)
C6—C5—C3	100.94 (17)	C16—C15—C14	119.7 (3)
C9—C5—Cl4	116.28 (16)	C15—C16—C17	120.2 (3)
C6—C5—Cl4	116.64 (17)	C15—C16—H16	119.9
C3—C5—Cl4	114.41 (15)	C17—C16—H16	119.9
C7—C6—C5	92.81 (17)	C12—C17—C16	121.1 (3)
C7—C6—Cl5	113.55 (16)	C12—C17—H17	119.4
C5—C6—Cl5	114.70 (15)	C16—C17—H17	119.4
C7—C6—Cl6	113.64 (16)	O3—C18—H18A	109.5
C5—C6—Cl6	113.18 (16)	O3—C18—H18B	109.5
Cl5—C6—Cl6	108.47 (12)	H18A—C18—H18B	109.5
C8—C7—C6	99.14 (18)	O3—C18—H18C	109.5
C8—C7—C2	107.55 (17)	H18A—C18—H18C	109.5
C6—C7—C2	100.98 (18)	H18B—C18—H18C	109.5
C8—C7—Cl1	115.47 (16)	O4—C19—H19A	109.5
C6—C7—Cl1	116.16 (16)	O4—C19—H19B	109.5
C2—C7—Cl1	115.42 (16)	H19A—C19—H19B	109.5
C9—C8—C7	107.41 (19)	O4—C19—H19C	109.5
C9—C8—Cl2	128.61 (18)	H19A—C19—H19C	109.5
C7—C8—Cl2	123.74 (17)	H19B—C19—H19C	109.5
C8—C9—C5	107.74 (19)		
			
C4—N1—C1—O1	−179.4 (2)	C1—C2—C7—C8	−47.0 (3)
C10—N1—C1—O1	2.1 (4)	C3—C2—C7—C8	66.0 (2)
C4—N1—C1—C2	2.3 (3)	C1—C2—C7—C6	−150.4 (2)
C10—N1—C1—C2	−176.2 (2)	C3—C2—C7—C6	−37.3 (2)
O1—C1—C2—C3	179.7 (2)	C1—C2—C7—Cl1	83.5 (2)
N1—C1—C2—C3	−2.1 (2)	C3—C2—C7—Cl1	−163.44 (16)
O1—C1—C2—C7	−68.6 (3)	C6—C7—C8—C9	34.4 (2)
N1—C1—C2—C7	109.7 (2)	C2—C7—C8—C9	−70.2 (2)
C1—C2—C3—C4	1.1 (2)	Cl1—C7—C8—C9	159.29 (17)
C7—C2—C3—C4	−117.95 (19)	C6—C7—C8—Cl2	−150.73 (16)
C1—C2—C3—C5	119.72 (18)	C2—C7—C8—Cl2	104.6 (2)
C7—C2—C3—C5	0.6 (2)	Cl1—C7—C8—Cl2	−25.9 (3)
C1—N1—C4—O2	178.5 (2)	C7—C8—C9—C5	0.7 (2)
C10—N1—C4—O2	−3.0 (4)	Cl2—C8—C9—C5	−173.78 (16)
C1—N1—C4—C3	−1.6 (3)	C7—C8—C9—Cl3	177.45 (17)
C10—N1—C4—C3	176.95 (19)	Cl2—C8—C9—Cl3	3.0 (3)
C2—C3—C4—O2	−180.0 (2)	C6—C5—C9—C8	−35.4 (2)
C5—C3—C4—O2	68.6 (3)	C3—C5—C9—C8	69.1 (2)
C2—C3—C4—N1	0.1 (2)	Cl4—C5—C9—C8	−161.17 (16)
C5—C3—C4—N1	−111.3 (2)	C6—C5—C9—Cl3	147.60 (17)
C4—C3—C5—C9	45.4 (3)	C3—C5—C9—Cl3	−107.85 (19)
C2—C3—C5—C9	−67.1 (2)	Cl4—C5—C9—Cl3	21.9 (2)
C4—C3—C5—C6	148.5 (2)	C1—N1—C10—C11	94.0 (3)
C2—C3—C5—C6	36.1 (2)	C4—N1—C10—C11	−84.4 (3)
C4—C3—C5—Cl4	−85.4 (2)	N1—C10—C11—C12	175.8 (3)
C2—C3—C5—Cl4	162.17 (16)	C10—C11—C12—C17	99.6 (4)
C9—C5—C6—C7	52.57 (18)	C10—C11—C12—C13	−82.5 (4)
C3—C5—C6—C7	−57.35 (18)	C17—C12—C13—C14	1.1 (4)
Cl4—C5—C6—C7	178.05 (15)	C11—C12—C13—C14	−176.8 (2)
C9—C5—C6—Cl5	170.14 (16)	C18—O3—C14—C13	−7.2 (4)
C3—C5—C6—Cl5	60.2 (2)	C18—O3—C14—C15	172.3 (3)
Cl4—C5—C6—Cl5	−64.4 (2)	C12—C13—C14—O3	−179.7 (2)
C9—C5—C6—Cl6	−64.7 (2)	C12—C13—C14—C15	0.8 (4)
C3—C5—C6—Cl6	−174.59 (15)	C19—O4—C15—C16	7.7 (4)
Cl4—C5—C6—Cl6	60.8 (2)	C19—O4—C15—C14	−172.6 (2)
C5—C6—C7—C8	−52.15 (18)	O3—C14—C15—O4	−1.2 (3)
Cl5—C6—C7—C8	−170.68 (14)	C13—C14—C15—O4	178.4 (2)
Cl6—C6—C7—C8	64.71 (19)	O3—C14—C15—C16	178.5 (2)
C5—C6—C7—C2	57.87 (18)	C13—C14—C15—C16	−1.9 (3)
Cl5—C6—C7—C2	−60.66 (19)	O4—C15—C16—C17	−179.2 (2)
Cl6—C6—C7—C2	174.73 (15)	C14—C15—C16—C17	1.2 (4)
C5—C6—C7—Cl1	−176.50 (15)	C13—C12—C17—C16	−1.9 (4)
Cl5—C6—C7—Cl1	65.0 (2)	C11—C12—C17—C16	176.0 (2)
Cl6—C6—C7—Cl1	−59.6 (2)	C15—C16—C17—C12	0.8 (4)

### Hydrogen-bond geometry (Å, º)

*Cg*5 is the centroid of the C12–C17 benzene ring.

**Table:**

*D*—H···*A*	*D*—H	H···*A*	*D*···*A*	*D*—H···*A*
C19—H19*A*···O2^i^	0.96	2.57	3.408 (4)	146
C6—Cl6···*Cg*5^ii^	1.77 (1)	3.41 (1)	4.894 (2)	140 (1)

Symmetry codes: (i) *y*+1/4, −*x*+3/4, −*z*+7/4; (ii) −*y*+3/4, *x*−1/4, −*z*+3/4.
