# Supplementary material for: Crystal structure of 4,5,6,7,8,8-hexa­chloro-2-(3,4-di­meth­oxy­pheneth­yl)-3a,4,7,7a-tetra­hydro-1H-4,7-methano­iso­indole-1,3(2H)-dione [+solvent]
Source: Acta Crystallogr E Crystallogr Commun. 2019 Apr 2;75(Pt 5):562–4. doi: 10.1107/S2056989019004109 (PMC6505607; doi:10.1107/S2056989019004109)
Supplement: Supplementary file 3 [file e-75-00562-sup4.pdf]

Search Overview

Search:	search2
Date/Time done:	Sat Mar 16 18:46:15 2019
Database(s):	CSD version 5.40 updates (Feb 2019)<br>CSD version 5.40 (November 2018)
Restriction Info:	No refcode restrictions applied
Filters:	None
Percentage Completed:	100%
Number of Hits:	17

Single query used. Search found structures that:

match

Query 1

Query 1

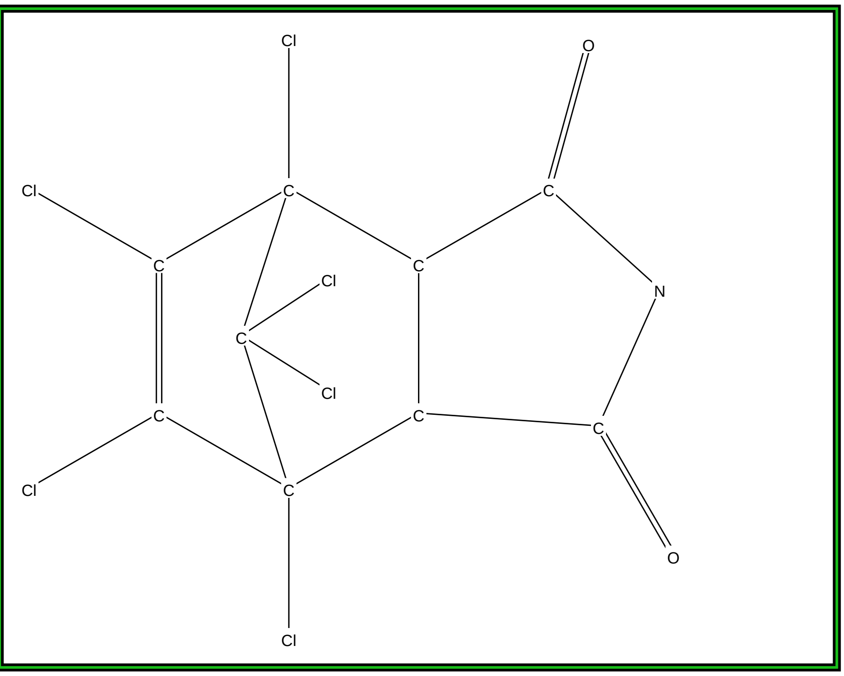

Search: search2 (Sat Mar 16 18:46:15 2019): Hits 1-4

AYUWAT

Reference: R.Manohar, M.Harikrishna, C.R.Ramanathan, M.SureshKumar, K.Gunasekaran (2011) *Acta Crystallogr.,Sect.E:Struct.Rep.Online* ,**67,**o2391

Formula: $C_{14} H_7 Cl_6 N_1 O_2 S_1$

Compound Name: 1,7,8,9,10,10-Hexachloro-4-(thiophen-2-ylmethyl)-4-azatricyclo[5.2.1.0^{2,6}]dec-8-ene-3,5-dione

Space Group:	I41/a	***Cell:***	***a*** 23.814(1)	***b*** 23.814(1)	***c*** 12.624(0)		
Space Group No.:	88	***(Å,°)***	α 90.00	β 90.00	γ 90.00		
R-Factor (%):	3.71	***Temperature(K):***	293	***Density(g/cm^3):***	1.729		

Parameters

Fragment 1

**DIST1 (D)**	1.689
**DIST2 (D)**	1.695
**DIST3 (D)**	1.741
**DIST4 (D)**	1.749
**DIST5 (D)**	1.763
**DIST6 (D)**	1.761

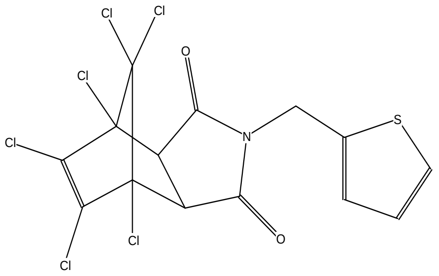

EVEDIT

Reference: R.Manohar, M.Harikrishna, C.R.Ramanathan, M.S.Kumar, K.Gunasekaran (2011) *Acta Crystallogr.,Sect.E:Struct.Rep.Online* ,**67,** o1708

Formula: $C_{17} H_{11} Cl_6 N_1 O_2$

Compound Name: 1,7,8,9,10,10-Hexachloro-4-(2-phenylethyl)-4-azatricyclo[5.2.1.0^{2,6}]dec-8-ene-3,5-dione

Space Group:	P21/c	***Cell:***	***a*** 13.301(0)	***b*** 13.614(0)	***c*** 11.491(0)		
Space Group No.:	14	***(Å,°)***	α 90.00	β 111.28(0)	γ 90.00		
R-Factor (%):	4.20	***Temperature(K):***	293	***Density(g/cm^3):***	1.624		

Parameters

Fragment 1

**DIST1 (D)**	1.688
**DIST2 (D)**	1.692
**DIST3 (D)**	1.751
**DIST4 (D)**	1.742
**DIST5 (D)**	1.770
**DIST6 (D)**	1.763

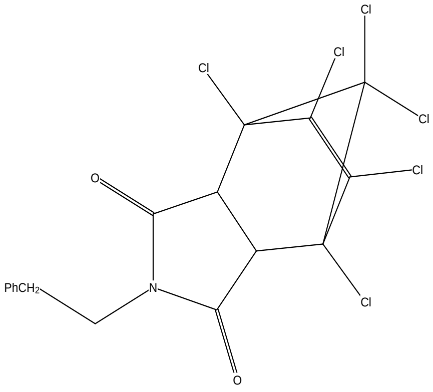

SUDJEI

Reference: J.Gebers, D.Rolland, R.Marty, S.Suarez, L.Cervini, R.Scopelliti, J.C.Brauer, H.Frauenrath (2015) *Chem.-Eur.J.* ,**21,**1542

Formula: $C_{20} H_{11} Cl_6 N_1 O_4 S_2$

Compound Name: 4-((3-(2,2'-Bithiophen-5-yl)propanoyl)oxy)-1,7,8,9,10,10-hexachloro-4-azatricyclo[5.2.1.0^{2,6}]dec-8-ene-3,5-dione

Space Group:	P21/c	***Cell:***	***a*** 14.738(1)	***b*** 11.558(2)	***c*** 14.031(1)		
Space Group No.:	14	***(Å,°)***	α 90.00	β 103.59(0)	γ 90.00		
R-Factor (%):	2.46	***Temperature(K):***	100	***Density(g/cm^3):***	1.733		

Parameters

Fragment 1

**DIST1 (D)**	1.700
**DIST2 (D)**	1.695
**DIST3 (D)**	1.749
**DIST4 (D)**	1.750
**DIST5 (D)**	1.774
**DIST6 (D)**	1.763

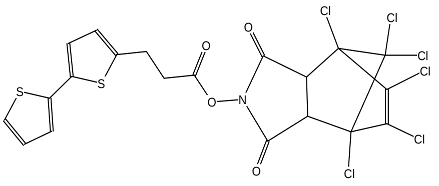

SUDJIM

Reference: J.Gebers, D.Rolland, R.Marty, S.Suarez, L.Cervini, R.Scopelliti, J.C.Brauer, H.Frauenrath (2015) *Chem.-Eur.J.* ,**21,**1542

Formula: $C_{20} H_{10} Br_1 Cl_6 N_1 O_4 S_2$

Compound Name: 4-((3-(5'-Bromo-2,2'-bithiophen-5-yl)propanoyl)oxy)-1,7,8,9,10,10-hexachloro-4-azatricyclo[5.2.1.0^{2,6}]dec-8-ene-3,5-dione

Space Group:	P21/c	***Cell:***	***a*** 14.515(2)	***b*** 11.643(1)	***c*** 14.884(0)		
Space Group No.:	14	***(Å,°)***	α 90.00	β 101.64(0)	γ 90.00		
R-Factor (%):	1.95	***Temperature(K):***	100	***Density(g/cm^3):***	1.847		

Parameters

Fragment 1

**DIST1 (D)**	1.705
**DIST2 (D)**	1.703
**DIST3 (D)**	1.757
**DIST4 (D)**	1.754
**DIST5 (D)**	1.776
**DIST6 (D)**	1.765

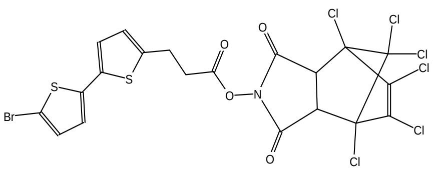

Search: search2 (Sat Mar 16 18:46:15 2019): Hits 5-8

SUDJOS

Reference: J.Gebers, D.Rolland, R.Marty, S.Suarez, L.Cervini, R.Scopelliti, J.C.Brauer, H.Frauenrath (2015) *Chem.-Eur.J.* ,**21,**1542

Formula: $C_9 H_3 Cl_6 N_1 O_3,2(H_2 O_1)$

Compound Name: 1,7,8,9,10,10-Hexachloro-4-hydroxy-4-azatricyclo[5.2.1.0^{2,6}]dec-8-ene-3,5-dione dihydrate

Synonym: Chlorendic hydroxylimide dihydrate

Space Group:	P21/c	***Cell:***	***a*** 15.037(4)	***b*** 8.258(2)	***c*** 12.556(5)
Space Group No.:	14	***(Å,°)***	α 90.00	β 108.88(1)	γ 90.00

R-Factor (%): 4.81 ***Temperature(K):*** 140 ***Density(g/cm^3):*** 1.899

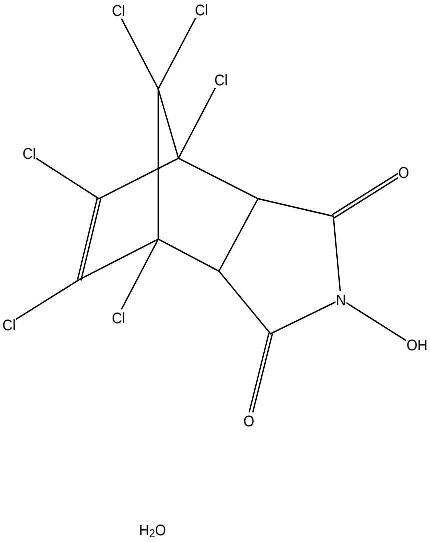

Parameters

Fragment 1	
**DIST1 (D)**	1.698
**DIST2 (D)**	1.695
**DIST3 (D)**	1.750
**DIST4 (D)**	1.746
**DIST5 (D)**	1.762
**DIST6 (D)**	1.769

SUDVEU

Reference: J.Gebers, D.Rolland, R.Marty, S.Suarez, L.Cervini, R.Scopelliti, J.C.Brauer, H.Frauenrath (2015) *Chem.-Eur.J.* ,**21,**1542

Formula: $C_{15} H_9 Cl_6 N_1 O_2 S_1$

Compound Name: 2-(2-(endo-1,7,8,9,10,10-Hexachloro-3,5-dioxo-4-aza-tricyclo[5.2.1.02,6]dec-8-en-4-yl)-ethyl)thiophene

Space Group:	P21/c	***Cell:***	***a*** 12.991(0)	***b*** 13.332(1)	***c*** 11.345(0)
Space Group No.:	14	***(Å,°)***	α 90.00	β 111.58(0)	γ 90.00

R-Factor (%): 2.74 ***Temperature(K):*** 100 ***Density(g/cm^3):*** 1.745

Parameters

Fragment 1	
**DIST1 (D)**	1.700
**DIST2 (D)**	1.706
**DIST3 (D)**	1.752
**DIST4 (D)**	1.758
**DIST5 (D)**	1.767
**DIST6 (D)**	1.780

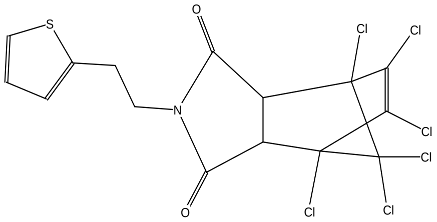

SUDVIY

Reference: J.Gebers, D.Rolland, R.Marty, S.Suarez, L.Cervini, R.Scopelliti, J.C.Brauer, H.Frauenrath (2015) *Chem.-Eur.J.* ,**21,**1542

Formula: $C_{19} H_{11} Cl_6 N_1 O_2 S_2$

Compound Name: 4-(2-(2,2'-Bithiophen-5-yl)ethyl)-1,7,8,9,10,10-hexachloro-4-azatricyclo[5.2.1.0^{2,6}]dec-8-ene-3,5-dione

Space Group:	P21/n	***Cell:***	***a*** 12.319(2)	***b*** 7.633(0)	***c*** 47.575(10)
Space Group No.:	14	***(Å,°)***	α 90.00	β 90.93(1)	γ 90.00

R-Factor (%): 9.23 ***Temperature(K):*** 100 ***Density(g/cm^3):*** 1.669

Parameters

Fragment 1	
**DIST1 (D)**	1.687
**DIST2 (D)**	1.712
**DIST3 (D)**	1.725
**DIST4 (D)**	1.748
**DIST5 (D)**	1.763
**DIST6 (D)**	1.768

Fragment 2	
**DIST1 (D)**	1.697
**DIST2 (D)**	1.687
**DIST3 (D)**	1.739
**DIST4 (D)**	1.756
**DIST5 (D)**	1.771
**DIST6 (D)**	1.773

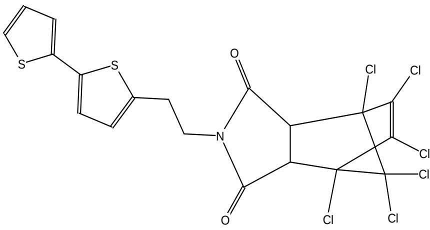

SUDVOE

Reference: J.Gebers, D.Rolland, R.Marty, S.Suarez, L.Cervini, R.Scopelliti, J.C.Brauer, H.Frauenrath (2015) *Chem.-Eur.J.* ,**21,**1542

Formula: $C_{19} H_{10} Br_1 Cl_6 N_1 O_2 S_2$

Compound Name: 4-(2-(5'-Bromo-2,2'-bithiophen-5-yl)ethyl)-1,7,8,9,10,10-hexachloro-4-azatricyclo[5.2.1.0^{2,6}]dec-8-ene-3,5-dione

Space Group:	P21/n	***Cell:***	***a*** 12.053(6)	***b*** 7.703(1)	***c*** 51.389(10)
Space Group No.:	14	***(Å,°)***	α 90.00	β 92.31(2)	γ 90.00

R-Factor (%): 8.34 ***Temperature(K):*** 100 ***Density(g/cm^3):*** 1.786

Parameters

Fragment 1	
**DIST1 (D)**	1.690
**DIST2 (D)**	1.699
**DIST3 (D)**	1.752
**DIST4 (D)**	1.745
**DIST5 (D)**	1.756
**DIST6 (D)**	1.768

Fragment 2	
**DIST1 (D)**	1.700
**DIST2 (D)**	1.692
**DIST3 (D)**	1.736
**DIST4 (D)**	1.753
**DIST5 (D)**	1.761
**DIST6 (D)**	1.779

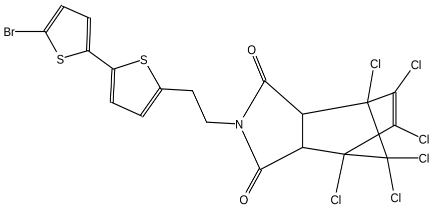

Search: search2 (Sat Mar 16 18:46:15 2019): Hits 9-12

SUDVUK

Reference: J.Gebers, D.Rolland, R.Marty, S.Suarez, L.Cervini, R.Scopelliti, J.C.Brauer, H.Frauenrath (2015) *Chem.-Eur.J.* ,**21,**1542

Formula: $C_{23} H_{13} Cl_6 N_1 O_2 S_3$

Compound Name: 1,7,8,9,10,10-Hexachloro-4-(2-(2,2':5',2''-terthiophen-5-yl)ethyl)-4-azatricyclo[5.2.1.$0^{2,6}$]dec-8-ene-3,5-dione

Space Group: P-1	***Cell:***	***a*** 7.822(1)	***b*** 12.068(1)	***c*** 27.956(6)
Space Group No.: 2	***(Å,°)***	α 80.55(1)	β 87.24(1)	γ 89.88(1)

R-Factor (%): 13.17 ***Temperature(K):*** 100 ***Density(g/cm³):*** 1.646

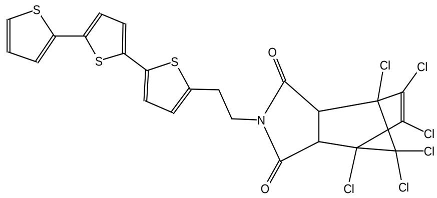

Parameters

Fragment 1	
**DIST1 (D)**	1.681
**DIST2 (D)**	1.705
**DIST3 (D)**	1.746
**DIST4 (D)**	1.736
**DIST5 (D)**	1.752
**DIST6 (D)**	1.766

Fragment 2	
**DIST1 (D)**	1.690
**DIST2 (D)**	1.690
**DIST3 (D)**	1.742
**DIST4 (D)**	1.766
**DIST5 (D)**	1.759
**DIST6 (D)**	1.770

SUDWAR

Reference: J.Gebers, D.Rolland, R.Marty, S.Suarez, L.Cervini, R.Scopelliti, J.C.Brauer, H.Frauenrath (2015) *Chem.-Eur.J.* ,**21,**1542

Formula: $C_{38} H_{20} Cl_{12} N_2 O_4 S_4$

Compound Name: 4,4'-(2,2':5',2'':5'',2'''-Quaterthiene-5,5'''-diyldiethane-2,1-diyl)bis(1,7,8,9,10,10-hexachloro-4-azatricyclo[5.2.1.$0^{2,6}$]dec-8-ene-3,5-dione)

Space Group: P-1	***Cell:***	***a*** 8.454(1)	***b*** 19.539(3)	***c*** 24.348(3)
Space Group No.: 2	***(Å,°)***	α 69.98(1)	β 86.89(1)	γ 80.47(1)

R-Factor (%): 9.52 ***Temperature(K):*** 100 ***Density(g/cm³):*** 1.500

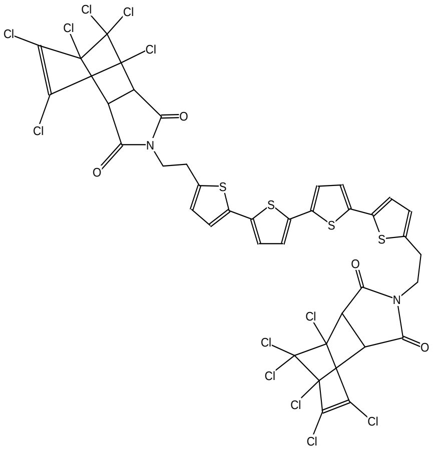

Parameters

Fragment 1	
**DIST1 (D)**	1.706
**DIST2 (D)**	1.700
**DIST3 (D)**	1.753
**DIST4 (D)**	1.762
**DIST5 (D)**	1.762
**DIST6 (D)**	1.787

Fragment 2	
**DIST1 (D)**	1.709
**DIST2 (D)**	1.687
**DIST3 (D)**	1.759
**DIST4 (D)**	1.746
**DIST5 (D)**	1.764
**DIST6 (D)**	1.768

Fragment 3	
**DIST1 (D)**	1.702
**DIST2 (D)**	1.735
**DIST3 (D)**	1.767
**DIST4 (D)**	1.756
**DIST5 (D)**	1.798
**DIST6 (D)**	1.748

SUDWEV

Reference: J.Gebers, D.Rolland, R.Marty, S.Suarez, L.Cervini, R.Scopelliti, J.C.Brauer, H.Frauenrath (2015) *Chem.-Eur.J.* ,**21,**1542

Formula: $C_{38} H_{20} Cl_{12} N_2 O_4 S_4,C_7 H_8$

Compound Name: 4,4'-(2,2':5',2'':5'',2'''-Quaterthiene-5,5'''-diyldiethane-2,1-diyl)bis(1,7,8,9,10,10-hexachloro-4-azatricyclo[5.2.1.$0^{2,6}$]dec-8-ene-3,5-dione) toluene solvate

Space Group: P-1	***Cell:***	***a*** 12.029(1)	***b*** 14.250(3)	***c*** 15.813(2)
Space Group No.: 2	***(Å,°)***	α 88.51(1)	β 69.00(1)	γ 76.70(1)

R-Factor (%): 5.31 ***Temperature(K):*** 100 ***Density(g/cm³):*** 1.641

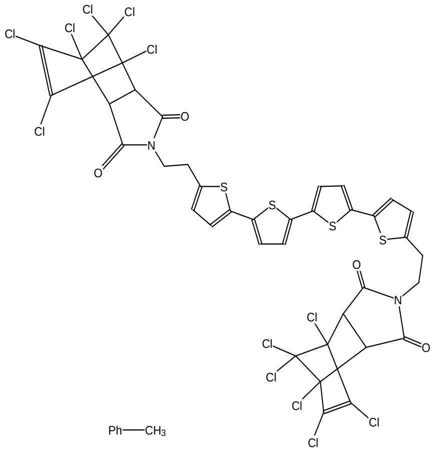

Parameters

Fragment 1	
**DIST1 (D)**	1.701
**DIST2 (D)**	1.703
**DIST3 (D)**	1.748
**DIST4 (D)**	1.748
**DIST5 (D)**	1.768
**DIST6 (D)**	1.773

Fragment 2	
**DIST1 (D)**	1.701
**DIST2 (D)**	1.703
**DIST3 (D)**	1.750
**DIST4 (D)**	1.755
**DIST5 (D)**	1.773
**DIST6 (D)**	1.771

SUDWIZ

Reference: J.Gebers, D.Rolland, R.Marty, S.Suarez, L.Cervini, R.Scopelliti, J.C.Brauer, H.Frauenrath (2015) *Chem.-Eur.J.* ,**21,**1542

Formula: $C_{46} H_{24} Cl_{12} N_2 O_4 S_6$

Compound Name: 4,4'-(2,2':5',2'':5'',2''':5''',2'''':5'''',2'''''-Sexithiene-5,5'''''-diyldiethane-2,1-diyl)bis(1,7,8,9,10,10-hexachloro-4-azatricyclo[5.2.1.$0^{2,6}$]dec-8-ene-3,5-dione)

Space Group: C2/c	***Cell:***	***a*** 45.229(9)	***b*** 8.119(1)	***c*** 14.175(3)
Space Group No.: 15	***(Å,°)***	α 90.00	β 102.10(3)	γ 90.00

R-Factor (%): 7.96 ***Temperature(K):*** 140 ***Density(g/cm³):*** 1.679

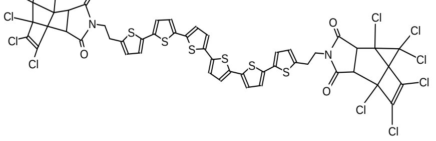

Parameters

Fragment 1	
**DIST1 (D)**	1.697
**DIST2 (D)**	1.701
**DIST3 (D)**	1.748
**DIST4 (D)**	1.748
**DIST5 (D)**	1.763
**DIST6 (D)**	1.756

Search: search2 (Sat Mar 16 18:46:15 2019): Hits 13-16

SUDWOF

Reference: J.Gebers, D.Rolland, R.Marty, S.Suarez, L.Cervini, R.Scopelliti, J.C.Brauer, H.Frauenrath (2015) *Chem.-Eur.J.* ,**21,**1542

Formula: $C_{15}\ H_8\ Br_1\ Cl_6\ N_1\ O_2\ S_1$

Compound Name: 2-(2-(endo-1,7,8,9,10,10-Hexachloro-3,5-dioxo-4-aza-tricyclo[5.2.1.02, 6]dec-8-en-4-yl)-ethyl)-5-bromothiophene

Space Group:	P21/c	***Cell:***	***a***	13.560(3)	***b***	18.723(4)	***c***	7.762(1)
Space Group No.:	14	***(Å,°)***	α	90.00	β	92.11(1)	γ	90.00

R-Factor (%): 14.06 ***Temperature(K):*** 100 ***Density(g/cm^3):*** 1.885

Parameters

Fragment 1

**DIST1 (D)**	1.717
**DIST2 (D)**	1.709
**DIST3 (D)**	1.758
**DIST4 (D)**	1.769
**DIST5 (D)**	1.760
**DIST6 (D)**	1.775

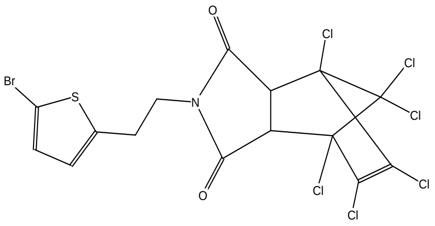

SUDWUL

Reference: J.Gebers, D.Rolland, R.Marty, S.Suarez, L.Cervini, R.Scopelliti, J.C.Brauer, H.Frauenrath (2015) *Chem.-Eur.J.* ,**21,**1542

Formula: $C_{16}\ H_9\ Cl_6\ N_1\ O_4\ S_1$

Compound Name: 1,7,8,9,10,10-Hexachloro-4-((3-(2-thienyl)propanoyl)oxy)-4-azatricyclo[5.2.1.0^{2,6}]dec-8-ene-3,5-dione

Space Group:	P21/c	***Cell:***	***a***	9.860(1)	***b***	14.586(3)	***c***	14.404(1)
Space Group No.:	14	***(Å,°)***	α	90.00	β	96.53(1)	γ	90.00

R-Factor (%): 4.57 ***Temperature(K):*** 100 ***Density(g/cm^3):*** 1.691

Parameters

Fragment 1

**DIST1 (D)**	1.703
**DIST2 (D)**	1.700
**DIST3 (D)**	1.749
**DIST4 (D)**	1.757
**DIST5 (D)**	1.777
**DIST6 (D)**	1.766

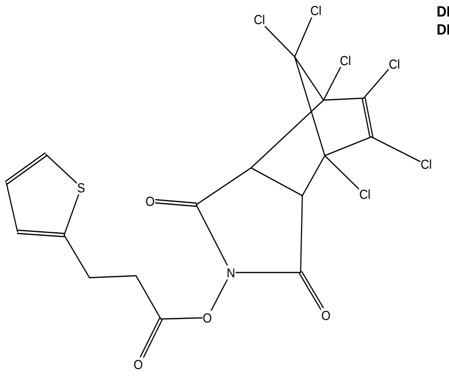

SUDXAS

Reference: J.Gebers, D.Rolland, R.Marty, S.Suarez, L.Cervini, R.Scopelliti, J.C.Brauer, H.Frauenrath (2015) *Chem.-Eur.J.* ,**21,**1542

Formula: $C_{16}\ H_8\ Br_1\ Cl_6\ N_1\ O_4\ S_1$

Compound Name: 4-((3-(5-bromo-2-thienyl)propanoyl)oxy)-1,7,8,9,10,10-hexachloro-4-azatricyclo[5.2.1.0^{2,6}]dec-8-ene-3,5-dione

Space Group:	P21/c	***Cell:***	***a***	13.654(0)	***b***	12.378(0)	***c***	12.858(0)
Space Group No.:	14	***(Å,°)***	α	90.00	β	95.33(0)	γ	90.00

R-Factor (%): 6.68 ***Temperature(K):*** 293 ***Density(g/cm^3):*** 1.851

Parameters

Fragment 1

**DIST1 (D)**	1.699
**DIST2 (D)**	1.693
**DIST3 (D)**	1.749
**DIST4 (D)**	1.745
**DIST5 (D)**	1.763
**DIST6 (D)**	1.763

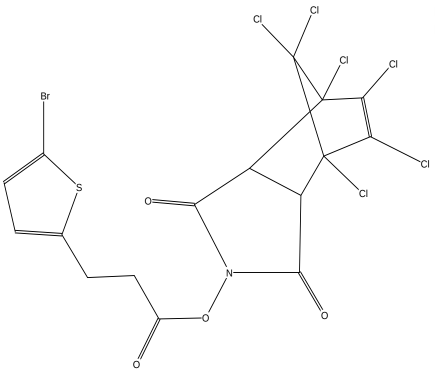

SUDXAS01

Reference: J.Gebers, D.Rolland, R.Marty, S.Suarez, L.Cervini, R.Scopelliti, J.C.Brauer, H.Frauenrath (2015) *Chem.-Eur.J.* ,**21,**1542

Formula: $C_{16}\ H_8\ Br_1\ Cl_6\ N_1\ O_4\ S_1$

Compound Name: 4-((3-(5-bromo-2-thienyl)propanoyl)oxy)-1,7,8,9,10,10-hexachloro-4-azatricyclo[5.2.1.0^{2,6}]dec-8-ene-3,5-dione

Space Group:	P21/n	***Cell:***	***a***	9.138(5)	***b***	14.011(6)	***c***	17.014(6)
Space Group No.:	14	***(Å,°)***	α	90.00	β	103.00(2)	γ	90.00

R-Factor (%): 4.56 ***Temperature(K):*** 140 ***Density(g/cm^3):*** 1.887

Parameters

Fragment 1

**DIST1 (D)**	1.698
**DIST2 (D)**	1.702
**DIST3 (D)**	1.747
**DIST4 (D)**	1.747
**DIST5 (D)**	1.760
**DIST6 (D)**	1.768

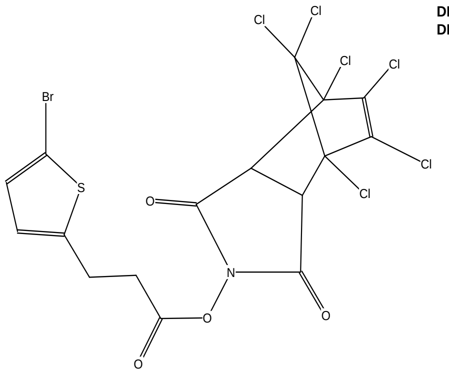

Search: search2 (Sat Mar 16 18:46:15 2019): Hit 17

TUDTUH

Reference:	B.Bartkowska, F.M.Bohnen, C.Kruger, W.F.Maier (1997)<br>*Acta Crystallogr.,Sect.C:Cryst.Struct.Commun.* ,**53,**521
Formula:	$C_{13} H_9 Cl_6 N_1 O_4,C_7 H_8$
Compound Name:	4-(1,7,8,9,10,10-Hexachloro-3,5-dioxo-4-aza-tricyclo(5.2.1.0^{2,6})dec-8-en-4-yl)butyric acid toluene solvate

Space Group:	P-1	***Cell:***	***a*** 8.766(2)	***b*** 10.759(2)	***c*** 13.158(2)
Space Group No.:	2	***(Å,°)***	α 105.08(1)	β 104.57(1)	γ 97.28(1)

R-Factor (%): 3.37 ***Temperature(K):*** 295 ***Density(g/cm^3):*** 1.604

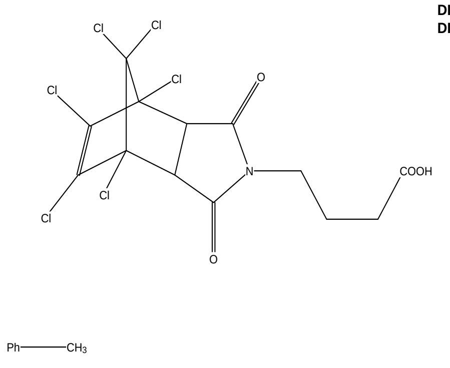

Parameters

Fragment 1

**DIST1 (D)**	1.691
**DIST2 (D)**	1.702
**DIST3 (D)**	1.751
**DIST4 (D)**	1.751
**DIST5 (D)**	1.763
**DIST6 (D)**	1.757
